# Bidirectional Association Between Premenstrual Disorders and Psychiatric Disorders

**DOI:** 10.1001/jamanetworkopen.2026.11765

**Published:** 2026-05-08

**Authors:** Jing Zhou, Zeinab Muse, Emma Bränn, Yihui Yang, Elgeta Hysaj, Miriam Martini, Nora E. Verberne, Marion Opatowski, Astrid Kamperman, Helena Kopp Kallner, Elizabeth Bertone-Johnson, Donghao Lu

**Affiliations:** 1 Institute of Environmental Medicine, Karolinska Institutet, Stockholm, Sweden; 2 Center for Epidemiology and Community Medicine, Region Stockholm, Stockholm, Sweden; 3 Department of Medical Epidemiology and Biostatistics, Karolinska Institutet, Stockholm, Sweden; 4 Department of Psychiatry, Erasmus University Medical Centre Rotterdam, Rotterdam, Netherlands; 5 Department of Clinical Sciences at Danderyd Hospital, Karolinska Institutet, Stockholm, Sweden; 6 Department of Obstetrics and Gynecology, Danderyd Hospital, Stockholm, Sweden; 7 Department of Biostatistics and Epidemiology, School of Public Health and Health Sciences, University of Massachusetts Amherst; 8 Department of Health Promotion and Policy, School of Public Health and Health Sciences, University of Massachusetts Amherst

## Abstract

**Importance:**

Premenstrual disorders (PMD) often co-occur with psychiatric conditions and exhibit overlapping symptoms. However, the direction and extent of this association remain poorly understood, particularly beyond major affective disorders.

**Objective:**

To investigate whether bidirectional associations exist between PMD and a broad range of psychiatric disorders and conditions.

**Design, Setting, and Participants:**

In this cohort study, Swedish nationwide and regional registers were used to identify women diagnosed with PMD from January 1, 2001, to December 31, 2022. Each identified woman was matched to their unaffected full sisters and to 10 unaffected controls. A nested case-control design was used to examine psychiatric disorders diagnosed prior to PMD, and a matched cohort design was used to assess incident psychiatric disorders occurring after PMD. The mean (SD) follow-up was 8.2 (5.8) years. Data were analyzed March 2025 to February 2026.

**Exposures:**

Clinical diagnosis of PMD and 14 subtypes of psychiatric disorders.

**Main Outcomes and Measures:**

Bidirectional associations were estimated using odds ratios (ORs) and hazard ratio (HRs) with 95% CIs.

**Results:**

Among 3 630 028 eligible women followed up for a mean (SD) of 8.2 (5.8) years, 104 972 were diagnosed with PMD (mean [SD] age, 35.4 [8.1] years). Of women with PMD, 50 176 (47.8%) had a previous psychiatric disorder diagnosis, compared with 309 802 (29.5%) of 1 049 720 unaffected controls, representing an approximately doubled risk (OR, 2.41 [95% CI, 2.38-2.44]). Similarly, women with PMD were twice as likely as women without PMD to receive a subsequent psychiatric disorder diagnosis (36.6% vs 21.1%; HR, 2.23 [95% CI, 2.19-2.27]). In sibling analyses, these bidirectional risks were attenuated but the associations remained (OR, 1.95 [95% CI, 1.89-2.01]; HR, 1.82 [95% CI, 1.74-1.90]). The highest bidirectional risks were for depression (OR, 2.19 [95% CI, 2.15-2.22]; HR, 2.70 [95% CI, 2.63-2.76]) and anxiety (OR, 2.26 [95% CI, 2.22-2.30]; HR, 2.43 [95% CI, 2.37-2.48]), with additional associations for attention-deficit/hyperactivity disorder (OR, 2.01 [95% CI, 1.94-2.09]; HR, 3.55 [95% CI, 3.32-3.80]), bipolar disorder (OR, 2.01 [95% CI, 1.93-2.10]; HR, 3.36 [95% CI, 3.07-3.67]), and personality disorder (OR, 2.01 [95% CI, 1.94-2.09]; HR, 3.34 [95% CI, 3.00-3.72]), but not for schizophrenia (OR, 1.01 [95% CI, 0.88-1.16]; HR, 1.00 [95% CI, 0.59-1.72]).

**Conclusions and Relevance:**

In this nationwide cohort study conducted in Sweden, bidirectional associations were found between PMD and psychiatric disorders and conditions, highlighting the need for sex- and menstrual cycle–informed care in psychiatry. Further research is needed to understand the underlying mechanisms shared between PMD and psychiatric disorders.

## Key Points

**Question:**

What are the risks of psychiatric disorders among women with premenstrual disorders (PMD) and vice versa?

**Findings:**

In a cohort study of 104 972 women with PMD and matched unaffected women, PMD were associated with an approximately 2-fold increased risk of developing psychiatric disorders, and psychiatric disorders were similarly associated with increased risk of subsequent PMD. The bidirectional risk was particularly pronounced for depression, anxiety, attention-deficit/hyperactivity disorder, bipolar disorder, and personality disorder.

**Meaning:**

These findings suggest that PMD and psychiatric disorders share bidirectional associations, highlighting the need for sex-specific and menstrual cycle–informed approaches in psychiatric assessment and care.

## Teaser

This nationwide cohort study investigates the risk of a broad range of psychiatric disorders or conditions among women with premenstrual disorders and the risk of subsequent premenstrual disorders among women with psychiatric disorders or conditions.

## Introduction

Premenstrual disorders (PMD), including premenstrual syndrome (PMS) and premenstrual dysphoric disorder, affect women of reproductive age with disabling symptoms, such as mood swings, anxiety, and irritability emerging during the luteal phase of the menstrual cycle.^1^ It is estimated that 20% to 30% of women are affected by moderate or severe PMS,^2,3^ whereas premenstrual dysphoric disorder affects 2% to 6% of women.^4,5^ Moreover, PMD are associated with a lowered quality of life,^6^ increased suicidal behavior,^7,8^ perinatal depression,^9^ early menopause and severe menopausal symptoms,^10^ and increased mortality among young patients.^11^

Evidence suggests that women with PMD have an atypical reaction to normal hormonal fluctuations in the menstrual cycle.^12,13^ This uncharacteristic reaction may contribute to psychiatric disorders through the influence of estrogen and progesterone on key neurotransmitter systems, including serotonin, γ-aminobutyric acid, and dopamine, which are also associated with certain psychiatric conditions.^1,3^ Genetic factors may also play a role, with twin and family studies showing an estimated heritability of 35% to 56% for PMD.^14^ Previous studies have also suggested a shared genetic landscape between PMD and major psychiatric disorders, including depression, bipolar disorder, attention-deficit/hyperactivity disorder (ADHD), schizophrenia, and autism.^15^ That said, the associations between PMD and psychiatric disorders may reflect both shared genetic predisposition and nongenetic, biological mechanisms.

A growing body of evidence suggests a bidirectional association between PMD and psychiatric disorders,^16^ most notably depression^17,18,19^ and anxiety^19,20^; women with PMD are 4 and 7 times more likely to have major depression and generalized anxiety disorders, respectively.^19,20^ Cross-sectional studies have also reported higher co-occurrence with bipolar disorder,^21,22^ posttraumatic stress disorder,^23,24^ and ADHD.^25^ However, small sample sizes, short follow-up periods, reliance on symptom-based assessments, and lack of prospective data limit exiting evidence. To date, only 2 prospective studies have reported an increased risk of depression and bipolar disorder following a PMD diagnosis,^22,26^ leaving the full spectrum of psychiatric co-occurrence unaddressed.

We therefore investigated the bidirectional association between PMD and psychiatric disorders in a nationwide register-based study in Sweden, together with a sibling comparison to account for early environmental and genetic factors shared between full sisters. We used the term *psychiatric disorder* as an inclusive category encompassing both psychiatric and neurodevelopmental conditions, acknowledging that definitions may vary across disciplines. We also used the term *women* to align with the literature, while recognizing that people may identify themselves differently.

## Methods

### Study Population

As described elsewhere,^9^ we conducted a nationwide nested case-control study and transitioned this design into a population-matched cohort study. Each population analysis was followed by a respective sibling comparison. The study was approved by the Swedish Ethical Review Authority. Informed consent is waived for register-based studies in Sweden. This study followed the Strengthening the Reporting of Observational Studies in Epidemiology (STROBE) reporting guideline.

We used multiple Swedish national registers, including the Total Population Register, National Patient Register (NPR), Prescribed Drug Register, Cause of Death Register, Medical Birth Register, and Longitudinal Integration Database for Health Insurance and Labor Market Studies. As PMD and psychiatric disorders are often diagnosed in primary care, we complemented our assessment using data from 5 primary care registers (Stockholm, Skåne, Västra Götaland, Uppsala, and Värmland), representing 60% of reproductive-age women in Sweden during the study period. All registers are cross-linked through the unique personal identification number. Details of registers are provided in the eMethods in Supplement 1.

**Figure 1.**
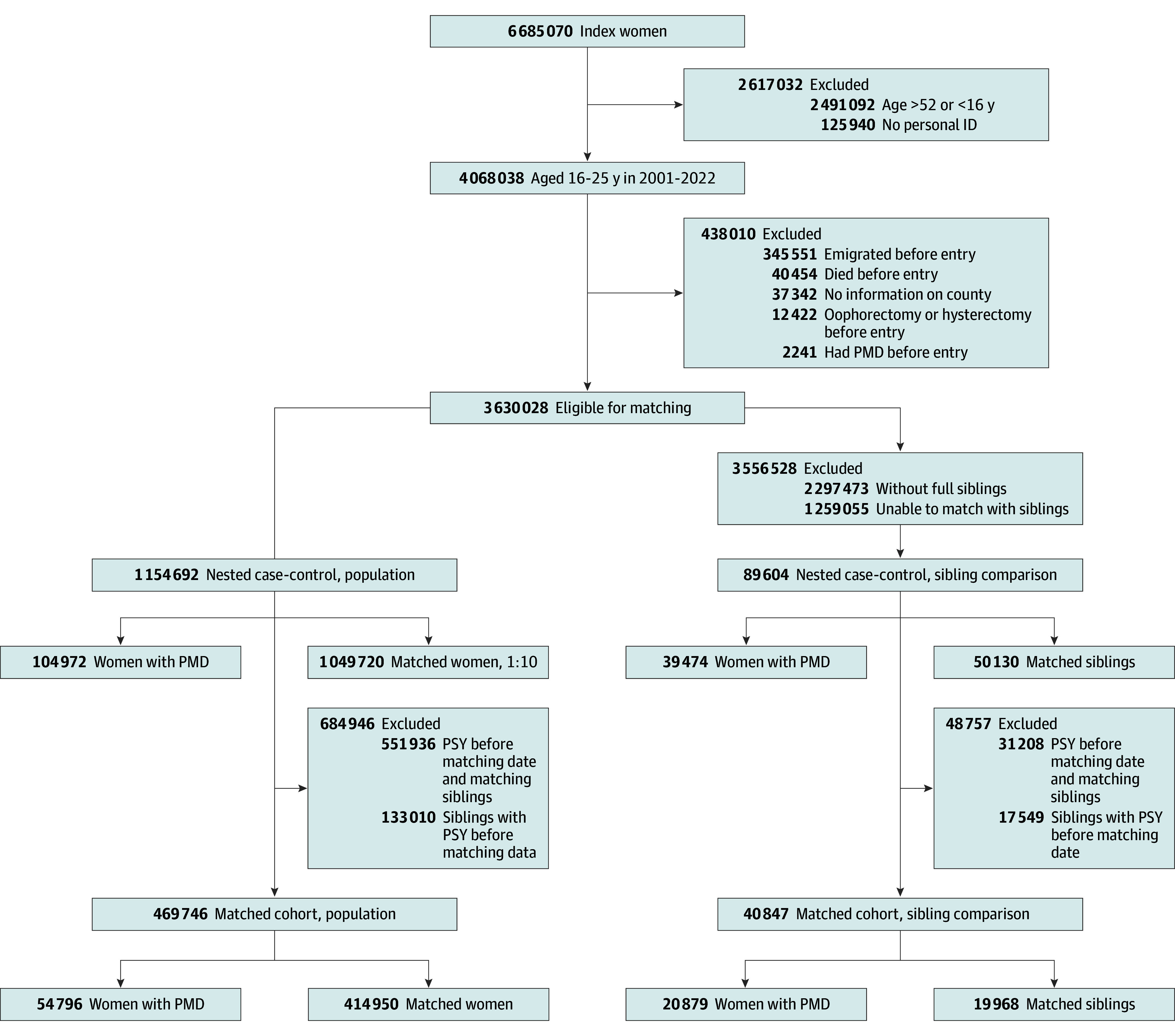
Flowchart for Population and Sibling Comparisons PMD indicates premenstrual disorders; PSY, psychiatric disorders.

As described elsewhere,^27^ all females aged 16 to 52 years from January 1, 2001, to December 31, 2022, were identified from the Total Population Register. We excluded women who lacked information on county of residence and women who died, emigrated, were diagnosed with PMD, or underwent bilateral oophorectomy or hysterectomy, whichever came later, leading to 3 630 028 eligible women in our study base (Figure 1).

### Ascertainment of PMD

In line with previous work by members of our team,^11^ we identified PMD cases through clinical diagnoses (codes provided in eTable 1 in Supplement 1) from the NPR and regional primary care registers. To complement the case identification outside the 5 counties with primary care data, we also identified prescriptions of antidepressants and contraceptives with a specified indication of PMD from the Prescribed Drug Register (eTable 1 in Supplement 1). Indications of PMD were specified by the prescribers as free-text and identified with key words recognition, as described elsewhere.^7^

### Ascertainment of Psychiatric Disorders

We identified any psychiatric diagnosis recorded in the NPR and regional primary care registers. Psychiatric disorders and conditions were classified into 14 subtypes: depressive disorders, anxiety disorders, stress-related disorders, schizophrenia, other psychotic disorders, bipolar disorder, eating disorder, alcohol use disorder, tobacco use disorder, other substance use disorders, ADHD, autism, behavioral disorders (such as conduct disorder), and personality disorders. The majority of these diagnoses have high validity.^28,29,30,31^ Overall, the positive predictive value of most diagnoses in the NPR ranges from 85% to 95%.^32^ Corresponding diagnosis codes are listed in eTable 1 in Supplement 1.

### Study Design

To investigate the association between psychiatric disorders and subsequent risk of PMD, we used a nested case-control study design. Using incidence density sampling, we randomly matched women with a first-ever PMD diagnosis (n = 104 972) to 10 unaffected women on year of birth and county of residence (n = 1 049 720) at the index date (the matching date). Continuous residency was not required prior to the index date. We then assessed any psychiatric diagnosis occurring before the matching date.

To examine the association between PMD and subsequent risk of psychiatric disorders, we conducted a matched cohort study. After excluding women with PMD and a prior psychiatric diagnosis and their matched unaffected women (n = 551 936), as well as unaffected women with prior psychiatric diagnoses (n = 133 010), a cohort of 54 796 women with PMD and 414 950 unaffected women was established for the prospective analysis (Figure 1). We followed up these women from the matching date until first diagnosis of a psychiatric condition, emigration, death, or December 31, 2022, and for unaffected women until a diagnosis of PMD if any (thereafter the woman contributed person-time to the PMD group), whichever came first.

Sibling comparisons were performed to address unmeasured confounders, such as shared genetic and childhood environmental factors.^33,34^ Specifically, we compared the odds and risks between women with PMD and their unaffected full sisters (ie, sharing 2 biological parents). For the association between a psychiatric condition and subsequent risk of PMD, we compared 39 474 women with PMD and their 50 130 unaffected sisters. For the association between PMD and subsequent risk of psychiatric conditions, we excluded women with PMD and a psychiatric diagnosis before the matching date, and their sisters, as well as unaffected sisters with a prior psychiatric diagnosis, yielding a cohort of 20 879 women with PMD and 19 968 unexposed siblings (Figure 1).

### Covariates

Demographic information at the matching date was obtained for calendar year, age, country of birth, county of residence, income, educational level, and civil (marital) status from registers. For parous women, information on body mass index (BMI) in early pregnancy and smoking 3 months before pregnancy was retrieved. All variables were categorized as detailed in eTable 2 in Supplement 1.

### Statistical Analysis

In the nested case-control study, conditional logistic regression was used to estimate odds ratios (ORs) and 95% CIs of subsequent PMD associated with psychiatric diagnoses, conditioning on the matching set. This design is analytically equivalent to a cohort study, and the estimated OR can be interpreted as a hazard ratio (HR).^35^ In the matched cohort study, stratified Cox regression (conditioning on the matching set with attained age as the underlying timescale) was used to estimate the HR of a psychiatric diagnosis following PMD. The assumption of proportional hazards was held over time according to the Schoenfeld residual.

We applied 2 models for both population and sibling analyses. Model 1 was adjusted for birth year and county of residence through conditioning on the matching set in the population analysis and through direct adjustment in the sibling analysis. Model 2 was additionally adjusted for country of birth, civil (marital) status, income, and educational level. Model 2 was considered the main model. Moreover, to provide insights into psychiatric subtypes, we analyzed type-specific psychiatric disorders in association with PMD.

We conducted several additional tests for the primary analysis (ie, PMD and any psychiatric disorder). To reduce risks of reverse causality and surveillance bias (ie, an early diagnosis of one disorder may lead to increased clinical attention to get the other diagnosis), we performed a period-specific analysis (≤5 and >5 years from the matching). Given that immigrants may face barriers to health care access and potential underdiagnoses, we performed stratified analysis by country of birth. To explore potential age-related differences in the associations between PMD and psychiatric disorder, we estimated the association for PMD diagnosed before and after the mean age (35 years). Due to the lack of nationwide primary care data, we restricted the analysis to the 5 counties with both primary care and specialist care data. Because obesity^36,37^ and smoking^38,39^ are associated with both PMD and psychiatric disorders, in an additional analysis restricting to parous women (due to data availability), we applied additional adjustments for BMI and smoking. To improve the validity of the PMD diagnosis, we restricted the analysis to women with at least 2 diagnoses more than 28 days apart.^11^ To address the possibility of surveillance bias, we conducted an additional analysis adjusting for the number of outpatient visits during the 6 months preceding the matching date as a proxy for recent health care utilization.

A 2-tailed *P* < .05 was considered statistically significant. Since multiple testing was not corrected for, *P* values should be interpreted as exploratory for the associations with type-specific psychiatric disorders. The data were prepared using SAS, version 9.4 (SAS Institute Inc) and analyzed in R, version 4.2.1 (R Project for Statistical Computing), from March 2025 to February 2026.

## Results

Among 3 630 028 eligible women, 104 972 were diagnosed with PMD (mean [SD] age at matching and diagnosis, 35.4 [8.1] years). Demographic characteristics are shown in eTable 2 in Supplement 1. Compared with women without PMD, women with PMD were more likely to be born in Scandinavia (86.3% vs 77.8%), be single (63.7% vs 61.4%), and have higher educational attainment (>12 years, 49.8% vs 45.9%) and annual household income (median [IQR] $2036 [$1505-$2725] vs $1931 [$1387-$2615]); similar patterns were found in the sibling comparison. Among parous women (43.1%), women with PMD were more likely than unaffected women (21.8% vs 19.7%) or sisters (21.5% vs 20.9%) to smoke 3 months prior to the latest pregnancy.

### Psychiatric Disorder and Subsequent Risk of PMD

A prior psychiatric diagnosis was found in 50 176 (47.8%) of women with PMD and 309 802 (29.5%) of women without PMD in the nested case-control study. After adjusting for demographics, we found that women with any psychiatric disorder had an increased risk of subsequent PMD (OR, 2.41 [95% CI, 2.38-2.44] (Table 1). In the sibling comparison, the OR was attenuated yet the association remained when comparing PMD cases with unaffected sisters (OR, 1.95 [95% CI, 1.89-2.01]) (Table 1).

**Table 1.** Association of Psychiatric Disorder With Subsequent Risk of PMD

Analysis	Total participants, No.	Psychiatric disorder, No. (%)	OR (95% CI)	
			Model 1^a^	Model 2^b^
**Population**				
No PMD	1 049 720	309 802 (29.5)	1 [Reference]	1 [Reference]
PMD	104 972	50 176 (47.8)	2.42 (2.39-2.45)	2.41 (2.38-2.44)
**Sibling**				
No PMD	50 130	17 549 (35.0)	1 [Reference]	1 [Reference]
PMD	39 474	18 595 (47.1)	1.91 (1.85-1.98)	1.95 (1.89-2.01)

Abbreviations: PMD, premenstrual disorder; OR, odds ratio.

^a^ Model 1 estimates were adjusted for the matching factors inherently controlled for through conditioning on the matching set.

^b^ Model 2 estimates were additionally adjusted for country of birth, civil (martial) status, income, and educational level.

### PMD and Subsequent Risk of Psychiatric Disorder

During a mean (SD) follow-up of 8.2 (5.8) years, 20 065 women with PMD (36.6%) and 87 409 unaffected women (21.1%) were diagnosed with a subsequent psychiatric disorder. In the population comparison, women with PMD had more than a 2-fold increased risk of a subsequent psychiatric disorder compared with unaffected women (HR, 2.23 [95% CI, 2.19-2.27]) (Table 2). An attenuated HR was observed in the sibling analysis although the association remained (HR, 1.82 [95% CI, 1.74-1.90]) (Table 2).

**Table 2.** Association of PMD With Subsequent Risk of Psychiatric Disorder

Analysis	Total participants, No.	Psychiatric disorder, No. (%)	HR (95% CI)	
			Model 1^a^	Model 2^b^
**Population**				
No PMD	414 950	87 409 (21.06)	1 [Reference]	1 [Reference]
PMD	54 796	20 065 (36.62)	2.21 (2.18-2.25)	2.23 (2.19-2.27)
**Sibling**				
No PMD	19 968	4650 (23.29)	1 [Reference]	1 [Reference]
PMD	20 879	7499 (35.92)	1.80 (1.72-1.88)	1.82 (1.74-1.90)

Abbreviations: HR, hazard ratio; PMD, premenstrual disorder.

^a^ Model 1 estimates were adjusted for the matching factors inherently controlled for through conditioning on the matching set.

^b^ Model 2 estimates were additionally adjusted for country of birth, civil (martial) status, income, and educational level.

We observed bidirectional associations for psychiatric disorders among parous women with PMD (n = 51 309) compared with matched counterparts (n = 446 471) (eTable 3 in Supplement 1). The results were largely unchanged when additionally adjusting for BMI and smoking among parous women.

### Type-Specific Psychiatric Disorder

**Figure 2.**
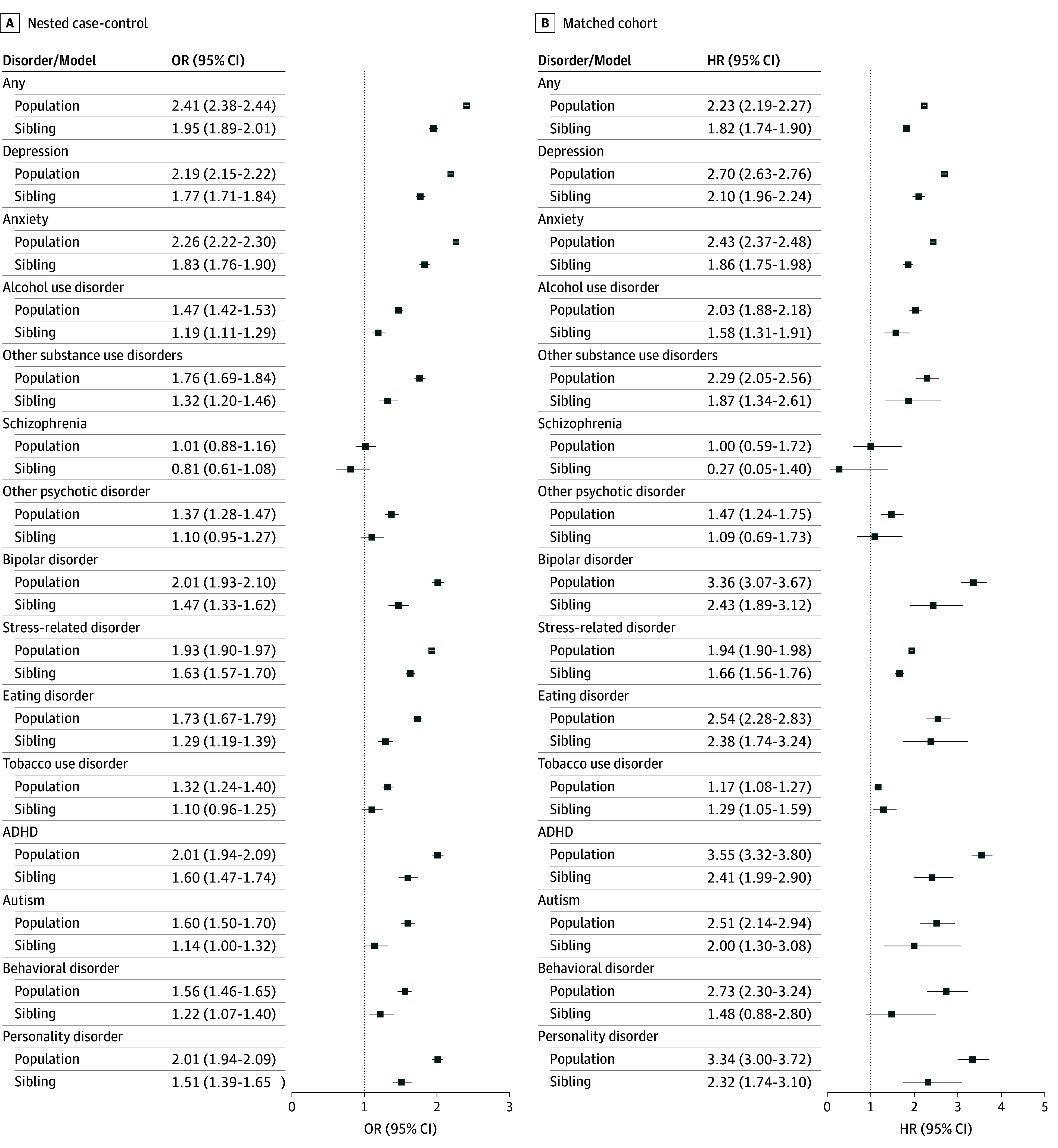
Dot-and-Whisker Plot of the Bidirectional Associations Between Premenstrual Disorders and Type-Specific Psychiatric Disorders All estimates were adjusted for matching factors, country of birth, civil (marital) status, income, and educational level. ADHD indicates attention-deficit/hyperactivity disorder; HR, hazard ratio; and OR, odds ratio.

We found a bidirectional association in 13 of 14 psychiatric disorders or conditions in the population analysis, and 11 of 14 in the sibling comparison (Figure 2; eTable 4 in Supplement 1). Notable associations included depression (nested case-control OR, 2.19 [95% CI, 2.15-2.22]; matched cohort HR, 2.70 [95% CI, 2.63-2.76]), anxiety (nested case-control OR, 2.26 [95% CI, 2.22-2.30]; matched cohort HR, 2.43 [95% CI, 2.37-2.48]), bipolar disorder (nested case-control OR, 2.01 [95% CI, 1.93-2.10]; matched cohort HR, 3.36 [95% CI, 3.07-3.67]), ADHD (nested case-control OR, 2.01 [95% CI, 1.94-2.09]; matched cohort HR, 3.55 [95% CI, 3.32-3.80]), autism (nested case-control OR, 1.60 [95% CI, 1.50-1.70]; matched cohort HR, 2.51 [95% CI, 2.14-2.94]), and personality disorders (nested case-control OR, 2.01 [95% CI, 1.94-2.09]; matched cohort HR, 3.34 [95% CI, 3.00-3.72]). There was no association between PMD and schizophrenia in either direction (nested case-control OR, 1.01 [95% CI, 0.88-1.16]; matched cohort HR, 1.00 [95% CI, 0.59-1.72]).

### Additional Analyses

In the period-specific analysis, the bidirectional association remained over time, with results somewhat attenuated for psychiatric disorders diagnosed more than 5 years after matching in the sibling comparison (eTable 5 in Supplement 1). Higher bidirectional risks were noted among women born outside Scandinavia and for women younger than 35 years at diagnosis or matching (eTable 6 in Supplement 1). The bidirectional associations between PMD and psychiatric disorders remained when restricting to counties with primary care data (eTable 7 in Supplement 1) or to women with 2 consecutive PMD diagnoses at least 28 days apart (eTable 8 in Supplement 1) and after additional adjustment for the number of outpatient visits during the 6 months preceding the index date (eTable 9 in Supplement 1).

## Discussion

In this nationwide, register-based study with 8.2 years of follow-up, women with psychiatric disorders or conditions were roughly twice as likely to later receive a PMD diagnosis. Women with PMD had an approximately doubled risk (HR, 2.23) of developing a subsequent psychiatric disorder or condition compared with their matched counterparts without PMD. When comparing with full sisters, the bidirectional associations remained although the hazard was attenuated (HR, 1.82). Notable bidirectional associations with PMD were observed for anxiety, depression, ADHD, bipolar disorder and personality disorders, whereas no association was observed for schizophrenia in either direction.

To our knowledge, no studies have examined the associations between PMD and the full spectrum of psychiatric conditions. The existing literature focuses on specific psychiatric disorders, particularly depression^18,26^ and anxiety.^20^ A small number of studies have also observed associations of PMD with posttraumatic stress disorder,^23,24^ substance use disorders,^40,41^ and eating disorders.^42^ Previous studies have mainly relied on PMD symptoms, as reported by surveys or short interviews,^18,20,23,42^ whereas our study assessed clinically diagnosed PMD using clinical data, thereby capturing a population with more severe and functionally impairing conditions. Furthermore, prior studies are limited by short follow-up periods, lack of prospective data, and small sample sizes. The bidirectional associations observed in our study, along with the largely consistent results in the sibling analyses, also align with previous studies, suggesting a genetic overlap between PMD and major psychiatric disorders.^15^

In the nested case-control study, the most notable associations included anxiety and depression, with these disorders also being the most prevalent in the study population. Compared with our findings, previous studies have reported higher ORs (eg, 4.76 for depression^19^ and 7.65^20^ for anxiety), which may stem from overestimation due to shared symptomology when measuring PMD and depression or anxiety symptoms at the same time. In a prospective study conducted in Taiwan, Li et al^22^ reported an HR of 2.58 for depression following a PMD diagnosis over a mean follow-up of 8 years. In line with that report, we also found that women with PMD were twice as likely to develop depression. Hypothalamic-pituitary-adrenal (HPA) axis dysregulation is a potential mechanism for PMD and mood and anxiety disorders, with research suggesting altered cortisol responses to stress and increased central nervous system sensitivity to hormonal fluctuations.^43,44,45,46^ Genetic factors also play a role, as indicated by prior research^15^ and by the attenuated risks observed in our sibling comparison.

Prior studies reported high comorbidity between PMD, bipolar disorder, and personality disorders.^21,47^ Our study is the first, to our knowledge, to illustrate bidirectional associations between PMD and these disorders. The cyclical affective symptoms of PMD can resemble features of bipolar or certain personality disorders, complicate differential diagnosis, and potentially lead to misclassification. PMD occurs during periods of pronounced reproductive hormonal fluctuations, which affect neurotransmitter systems such as dopamine, serotonin and γ-aminobutyric acid.^1,3^ These hormonal and neurochemical changes may disrupt mood regulation, increasing vulnerability to hypomanic or manic symptoms. Shared HPA axis dysregulation with heightened stress reactivity^43,48^ and shared genetic landscape^15^ may also contribute to this link.

Regarding neurodevelopmental conditions, we observed bidirectional associations between PMD and autism or ADHD. Women often experience delays in diagnosis of autism and ADHD,^49^ which may partly explain the increased detection of these conditions after a PMD diagnosis. In addition, sex hormones modulate dopamine signaling, which is reduced across several brain regions in women with autism and ADHD,^50,51^ potentially contributing to the shared neurobiological pathways.

Evidence on the association with schizophrenia is scarce and primarily based on small clinical samples. In a study of 50 inpatients with schizophrenia conducted in China, 52% reported premenstrual dysphoric disorder symptoms and 20% reported PMS symptoms.^52^ While genetic overlap between schizophrenia and PMD has been proposed,^15^ we did not observe an association in either direction. One explanation may be diagnostic overshadowing, in which the severe symptoms of schizophrenia eclipse other clinical concerns, such as PMD, leading to substantial underdiagnosis. Other factors include irregular cycles (thus masking symptoms) or amenorrhea resulting from the off-target effects of antipsychotic medication.^53^ However, these reasons may not explain the null association between PMD and subsequent schizophrenia, which warrants future investigations to better understand.

In addition to the aforementioned biological mechanisms, alternative explanations may contribute to our findings. PMD and several psychiatric disorders share overlapping symptoms (eg, irritability), which may lead to misclassification, although our use of register-based clinical diagnoses, rather than self-reported symptoms, reduces this concern. Moreover, PMD and psychiatric disorders share certain risk factors, such as smoking and obesity. Yet in a subgroup of parous women, the associations remained with comparable risks after adjustment for these factors assessed in the latest pregnancy. Shared psychosocial determinants, such as early-life adversity (eg, childhood maltreatment), may further contribute, yet the persistence of associations in sibling comparisons suggests that such shared familial factors alone are unlikely to fully explain our findings.

### Strengths and Limitations

One strength is that this nationwide, register-based study includes a large sample of women across all reproductive ages with comprehensive data, allowing for the examination of associations for less common psychiatric disorders that smaller studies cannot capture. However, several limitations should also be noted. First, the gold standard of PMD diagnosis relies on prospective daily symptoms ratings for a minimum of 2 consecutive menstrual cycles, which are not confirmed in registers.^54^ Nonetheless, the NPR generally demonstrates high validity, including for gynecological or psychiatric diagnoses.^32^ Our sensitivity analysis, restricting cases to women with at least 2 diagnoses more than 28 days apart, yielded similar results. Second, we studied psychiatric disorders occurring at any time during follow-up, without restricting to reproductive phases that are subject to hormone fluctuations. Future studies are needed to examine if such transitions (eg, perinatal and perimenopausal periods) constitute periods of specifically elevated risk. Third, diagnostic delays for both PMD and psychiatric disorders could affect observed temporal associations, although the consistent bidirectional associations observed more than 5 years before or after the index date argue against this notion. Finally, our findings reflect associations based on the timing of a clinical diagnosis rather than symptom onset. Because many psychiatric disorders manifested before the mean age of PMD diagnosis in our cohort, there is a possibility that some conditions (eg, bipolar disorder) were underrepresented in the analysis of incident psychiatric disorder following PMD. Future research examining the risk of psychiatric episodes or relapse may better capture the burden of mental health among women with PMD. Furthermore, while the bidirectional nature of these associations was robust, they should be interpreted as evidence of shared pathophysiological pathways rather than definitive causal relationships.

## Conclusions

In this nationwide cohort study conducted in Sweden, bidirectional associations were found between PMD and major psychiatric disorders or conditions, particularly depression, anxiety, ADHD, bipolar disorder, and personality disorder. Our findings highlight the need for raising awareness among health care providers on the higher risk of co-occurrence between these conditions and for providing sex-specific and menstrual cycle–informed care in psychiatry. Further research is needed to understand the underlying mechanisms shared between PMD and psychiatric disorders or conditions to develop novel therapeutic targets and refine treatment options across menstrual phases.

## Notes

Supplement 1.
eMethods.
**eTable 1.** International Classification of Diseases Codes and Anatomical Therapeutic Chemical Classification used in the study **eTable 2.** Characteristics of women with and without premenstrual disorders (PMD) **eTable 3.** Bidirectional associations between PMD and any psychiatric disorder in parous women, with additional adjustment for body mass index and smoking **eTable 4.** Bidirectional association between premenstrual disorders and type-specific psychiatric disorder **eTable 5.** Associations between (a) psychiatric disorders and risk of subsequent PMD and (b) PMD and subsequent risk of psychiatric disorders, by time of psychiatric disorders to/from PMD **eTable 6.** Bidirectional associations between premenstrual disorders and psychiatric disorders, stratified by country of birth and age at matching **eTable 7.** Associations between (a) psychiatric disorders and risk of subsequent PMD, and (b) PMD and subsequent risk of psychiatric disorders, restricting to counties with primary care data **eTable 8.** Associations between (a) psychiatric disorders and risk of subsequent PMD, and (b) PMD and subsequent risk of psychiatric disorders, restricting to women who receive consecutive PMD diagnoses which are at least 28 days apart **eTable 9.** Bidirectional association between premenstrual disorders and any psychiatric disorder, with additional adjustment for number of outpatient visits within 6 months before the matching date

Supplement 2.
Data Sharing Statement
