# Error in Abstract

**DOI:** 10.1001/jamanetworkopen.2026.25019

**Published:** 2026-06-29

The Original Investigation titled “Bidirectional Association Between Premenstrual Disorders and Psychiatric Disorders,”^1^ published on May 8, 2026, was corrected to fix wording in the Abstract Results, which now states that women with PMD were twice as likely as women without PMD to receive a subsequent psychiatric disorder diagnosis. This article was corrected online.^1^
