# Supplementary material for: Bidirectional Association Between Premenstrual Disorders and Psychiatric Disorders
Source: JAMA Netw Open. 2026 May 8;9(5):e2611765. doi: 10.1001/jamanetworkopen.2026.11765 (PMC13156785; doi:10.1001/jamanetworkopen.2026.11765)
Supplement: Supplement 1. — eMethods. eTable 1. International Classification of Diseases Codes and Anatomical Therapeutic Chemical Classification used in the study eTable 2. Characteristics of women with and without premenstrual disorders (PMD) eTable 3. Bidirectional associations between PMD and any psychiatric disorder in parous women, with additional adjustment for body mass index and smoking eTable 4. Bidirectional association between premenstrual disorders and type-specific psychiatric disorder eTable 5. Associations between (a) psychiatric disorders and risk of subsequent PMD and (b) PMD and subsequent risk of psychiatric disorders, by time of psychiatric disorders to/from PMD eTable 6. Bidirectional associations between premenstrual disorders and psychiatric disorders, stratified by country of birth and age at matching eTable 7. Associations between (a) psychiatric disorders and risk of subsequent PMD, and (b) PMD and subsequent risk of psychiatric disorders, restricting to counties with primary care data eTable 8. Associations between (a) psychiatric disorders and risk of subsequent PMD, and (b) PMD and subsequent risk of psychiatric disorders, restricting to women who receive consecutive PMD diagnoses which are at least 28 days apart eTable 9. Bidirectional association between premenstrual disorders and any psychiatric disorder, with additional adjustment for number of outpatient visits within 6 months before the matching date [file jamanetwopen-e2611765-s001.pdf]

Supplementary Online Content

Zhou J, Muse Z, Bränn E, et al. Bidirectional association between premenstrual disorders and psychiatric disorders. *JAMA Netw Open*. 2026;9(5):e2611765.
doi:10.1001/jamanetworkopen.2026.11765

eMethods.
eReferences.
eTable 1. International Classification of Diseases Codes and Anatomical Therapeutic Chemical Classification used in the study
eTable 2. Characteristics of women with and without premenstrual disorders (PMD)
eTable 3. Bidirectional associations between PMD and any psychiatric disorder in parous women, with additional adjustment for body mass index and smoking
eTable 4. Bidirectional association between premenstrual disorders and type-specific psychiatric disorder
eTable 5. Associations between (a) psychiatric disorders and risk of subsequent PMD and (b) PMD and subsequent risk of psychiatric disorders, by time of psychiatric disorders to/from PMD
eTable 6. Bidirectional associations between premenstrual disorders and psychiatric disorders, stratified by country of birth and age at matching
eTable 7. Associations between (a) psychiatric disorders and risk of subsequent PMD, and (b) PMD and subsequent risk of psychiatric disorders, restricting to counties with primary care data
eTable 8. Associations between (a) psychiatric disorders and risk of subsequent PMD, and (b) PMD and subsequent risk of psychiatric disorders, restricting to women who receive consecutive PMD diagnoses which are at least 28 days apart
eTable 9. Bidirectional association between premenstrual disorders and any psychiatric disorder, with additional adjustment for number of outpatient visits within 6 months before the matching date

This supplementary material has been provided by the authors to give readers additional information about their work.

© 2026 Zhou J et al. *JAMA Network Open*.

eMethods.

The Swedish national registers : (1) the Total Population Register (TPR)[1], which contains demographic data for all Swedish residents; (2) the National Patient Register (NPR)[2], which contains all hospital discharge diagnosis from 1987 onwards, and >80% hospital-based outpatient visits since 2001; (3) the Prescribed Drug Register (PDR)[3], which captures redeemed drug prescriptions from all pharmacies since July 2005; (4) the Longitudinal Integration Database for Health Insurance and Labor Market Studies (LISA), which integrates sociodemographic information for residents aged 16 and older since 1990; (5) the National Cause of Death Register, which comprises death certificates since 1952; (6) the Multi-Generation Register, which records parental information on residents since 1961; (7) the Medical Birth Register (MBR)[4], which contains data on antenatal care, delivery and neonatal characteristics for all births in Sweden from 1973 onwards.

The primary cares: (1) The Region Stockholm collects information from both public and private healthcare services in Stockholm County since 2001[5]. (2) The Skåne Healthcare Register retreived information on public and private healthcare consultations in Skåne county from 2001 onwards [6]. (3) The Region Västra Götaland (VGR) contains healthcare information from public and private healthcares for residents who have resgistered in the county since 2003[7]. (4) The Region Uppsala collects healthcare data from public healthcare providers since 2005[8]. (5) The Region Värmland owns information on only public health administration since 2014[9].

eReferences.

1. Ludvigsson JF, Almqvist C, Bonamy AK, et al. Registers of the Swedish total population and their use in medical research. *Eur J Epidemiol*. 2016;31:125-136.
2. Ludvigsson JF, Andersson E, Ekbom A, et al. External review and validation of the Swedish national inpatient register. *BMC Public Health*. 2011;11:450.
3. Wettermark B, Hammar N, Fored CM, et al. The new Swedish prescribed drug register: opportunities for pharmacoepidemiological research and experience from the first 6 months. *Pharmacoepidemiol Drug Saf*. 2007;16:726-735.
4. Ludvigsson JF, Andersson E, Ekbom A, et al. External review and validation of the Swedish national inpatient register. *BMC Public Health*. 2011;11. http://www.socialstyrelsen.se/Lists/Artikelkatalog/Attach ments/10655/2003-112-3_20031123.pdf. Accessed April 29, 2016.
5. Wändell P, Carlsson AC, Wettermark B, Lord G. Most common diseases diagnosed in primary care in Stockholm, Sweden, in 2011. 2013:506-513.
6. Löfvendahl S, Schelin ME, Jöud A. The value of the Skåne health-care register: prospectively collected individual-level data for population-based studies. *Scand J Public Health*. 2020;48:56-63.
7. Ödesjö H. Register-based evaluation of primary care: focus on chronic disease. 2019.
8. Care WP, Science M, Uppsala R. Research strategy for healthcare in region Uppsala 2021-2024. 2024;1:1-8.
9. Kirkinen T, Naimi-Akbar A, Cederlund A, et al. Accuracy of the Swedish quality registry for caries and periodontal diseases (SKaPa): evaluation in 6- and 12-year-olds in the region of Värmland, Sweden. *Acta Odontol Scand*. 2023;81:615-621.

© 2026 Zhou J et al. *JAMA Network Open*.

eTable 1. International Classification of Diseases Codes and Anatomical Therapeutic Chemical Classification used in the study

	ICD-9	ICD-10
**Premenstrual disorders**	625E	N943
ATC codes of antidepressants		N06AA, N06AB, N06AX
ATC codes of contraceptives		G02B, G03A
**Psychiatric disorders**	**ICD-9**	**ICD-10**
Depression	296.2x- 296.3x	F32-F33
Anxiety	300.00	F41
Alcohol use disorder	303.xx	F10
Other substance use disorders	304.0x-304.3x, 304.5x-304.6x, 304.8x, 305.7	F11-F16, F18-19
Schizophrenia	295.xx	F20
Other psychotic disorders	301.22, 297.1, 298.8, 297.3, 295.7, 298.9	F21-F25, F28-F29
Bipolar disorder	296.0x, 296.4x	F30-F31
Stress-related disorder	308.xx	F43
Eating disorder	307.1x	F50
Tobacco use disorder	305.1	F17
Attention deficit/hyperactivity disorder (ADHD)	314.0x	F90
Autism	299.xx	F84
Behavioral disorder	312.xx, 312.8, 313.1, 313.9, 307.20	F91-F98
Personality disorder	301.xx, 301.2x, 301.9	F60-F62, F69

ICD= International Classification of Diseases, ATC= Anatomical Therapeutic Chemical Classification.

© 2026 Zhou J et al. *JAMA Network Open*.

eTable 2. Characteristics of women with and without premenstrual disorders (PMD)

	Population comparison		Sibling comparison	
	Women with PMD	Women without PMD*	Women with PMD	Sisters without PMD
N	104,972	1,049,720	39,474	50,130
**At matching**				
**Age, mean (SD)**	35.4±8.1	35.4±8.1	34.9±7.7	34.7±8.6
**Age group**				
<30 years	28,213 (26.9)	282,185 (26.9)	11,269 (28.5)	15,925 (31.8)
30–39 years	43,325 (41.3)	432,738 (41.2)	16,972 (43.0)	19,179 (38.3)
≥40 years	33,434 (31.9)	334,797 (31.9)	11,233 (28.5)	15,026 (30.0)
**Country of birth (%)**				
Scandinavian	90,573 (86.3)	816,873 (77.8)	37,603 (95.3)	47,312 (94.4)
Other	14,399 (13.7)	232,847 (22.2)	1,871 (4.7)	2,818 (5.6)
**Region of residence**				
South	20,314 (19.4)	203,140 (19.4)	7,877 (20.0)	10,395 (20.7)
Middle	69,239 (66.0)	692,390 (66.0)	25,459 (64.5)	31,456 (62.7)
North	15,419 (14.7)	154,190 (14.7)	6,138 (15.5)	8,279 (16.5)
**Educational level**				
≤9	9,638 (9.2)	116,556 (11.1)	3,199 (8.1)	5,094 (10.2)
10 to 12	40,829 (38.9)	406,593 (38.7)	15,972 (40.5)	20,869 (41.6)
>12	52,322 (49.8)	481,493 (45.9)	19,938 (50.5)	23,445 (46.8)
**Individual household Income**				
median (IQR)	2,036 (1,505-2,725)	1,931(1,387-2,615)	2,060(1,543-2,731)	2,021(1,507-2,676)
Q1(Low)	21,395 (20.4)	265,563 (25.3)	9,500 (24.1)	12,888 (25.7)
Q2-Q3(Middle)	54,364 (51.8)	518,823 (49.4)	19,801 (50.2)	24,987 (49.8)
Q4(High)	28,981 (27.6)	257,581 (24.5)	10,160 (25.7)	12,205 (24.3)
Unknown	232 (0.2)	7753 (0.7)	13 (0.0)	50 (0.1)
**Civil status**				
Married/cohabited	37,931 (36.1)	398,885 (38.0)	13,809 (35.0)	17,121 (34.2)
Not married/cohabited	66,872 (63.7)	644,082 (61.4)	25,663 (65.0)	33,007 (65.8)
Unknown	169 (0.2)	6,753 (0.6)	2 (0.0)	2 (0.0)
**Women who have records in the Medical Birth Register**				
	Women with PMD	Matched women without PMD*	Women with PMD	Sisters without PMD
N	51,309	446,471	19,721	21,489
**Early BMI (kg/m^2) at the latest pregnancy**				
<18.5	769 (1.5)	8,387 (1.9)	293 (1.5)	332 (1.5)
18.5–24.9	29,617 (57.7)	245,521 (55.0)	11,542 (58.5)	12,216 (56.8)
25–29.9	12,915 (25.2)	112,218 (25.1)	4,861 (24.6)	5,211 (24.2)
≥30	5,581 (10.9)	58,561 (13.1)	2,152 (10.9)	2,650 (12.3)
Unknown	2,427 (4.7)	21,784 (4.9)	873 (4.4)	1,080 (5.0)
**Smoking three months prior to latest pregnancy (%)**				

© 2026 Zhou J et al. *JAMA Network Open*.

No	38,838 (75.7)	347,231 (77.8)	15,025 (76.2)	16,381 (76.2)
Yes	11,185 (21.8)	87,765 (19.7)	4,241 (21.5)	4,500 (20.9)
Unknown	1,286 (2.5)	11,475 (2.6)	455 (2.3)	608 (2.8)

PMD = Premenstrual disorders, SD = standard deviation. *Women matched to PMD women at the diagnosis date of PMD.

© 2026 Zhou J et al. *JAMA Network Open*.

eTable 3. Bidirectional associations between PMD and any psychiatric disorder in parous women, with additional adjustment for body mass index and smoking

(a)Nested-case control/From psychiatric disorder to PMD					(b) Matched-cohort/From PMD to psychiatric disorder				
**Population**					**Population**				
			Model1,	Model2,				Model1,	Model2,
	n	Psychiatric disorder, n (%)	OR (95%CI)	OR (95%CI)		n	Psychiatric disorder, n (%)	HR (95%CI)	HR (95%CI)
No PMD	446471	146203(32.75)	Ref.	Ref.	No PMD	206474	34411(16.67)	Ref.	Ref.
PMD	51309	25897(50.47)	2.33(2.28-2.38)	2.33(2.28-2.38)	PMD	25412	7325(28.82)	2.29 (2.22-2.37)	2.29(2.22-2.37)
**Sibling**					**Sibling**				
	n	Psychiatric disorder, n (%)	OR (95%CI)	OR (95%CI)		n	Psychiatric disorder, n (%)	HR (95%CI)	HR (95%CI)
No PMD	21489	8105(37.72)	Ref.	Ref.	No PMD	9637	1788(18.55)	Ref.	Ref.
PMD	19721	9695(49.16)	1.81(1.71-1.92)	1.82(1.72-1.93)	PMD	10026	2807(28.00)	1.79(1.61-1.99)	1.82(1.63-2.03)

PMD = Premenstrual disorders, OR = Odds ratio, HR = Hazard ratio, CI = Confidence interval, Ref = Reference.

Model1: The estimates were adjusted for matching variables, country of birth, civil status, income, and educational level.

Model2: The estimates were additionally adjusted for early pregnancy body mass index and smoking status prior to pregnancy.

© 2026 Zhou J et al. *JAMA Network Open*.

eTable 4. Bidirectional association between premenstrual disorders and type-specific psychiatric disorder

	(a)Nested-case control/From psychiatric disorder to PMD						(b)Matched-cohort/From PMD to psychiatric disorder					
	Population			Sibling			Population			Sibling		
	No PMD	PMD	Model (OR,95%CI)	Sisters	PMD	Model (OR,95%CI)	No PMD	PMD	Model (HR,95%CI)	Sisters	PMD	Model (HR,95%CI)
	PSY, n(%)	PSY, n(%)		PSY, n(%)	PSY, n(%)		PSY, n(%)	PSY, n(%)		PSY, n(%)	PSY, n(%)	
**Depression**	135,849 (12.94)	25,204(24.01)	2.19(2.15-2.22)	8,166(16.29)	9,350(23.69)	1.77(1.71-1.84)	31,425(7.57)	9,447(17.24)	2.70(2.63-2.76)	1,773(8.88)	3,549(17.00)	2.10(1.96-2.24)
**Anxiety**	138,255 (13.17)	25,714(24.5)	2.26(2.22-2.30)	8,269(16.50)	9,579(24.27)	1.83(1.76-1.90)	37,351(9)	10,543(19.24)	2.43(2.37-2.48)	2,094(10.49)	3,917(18.76)	1.86(1.75-1.98)
**Alcohol use disorder**	26,580(2.53)	4,001(3.81)	1.47(1.42-1.53)	1,656(3.30)	1,481(3.75)	1.19(1.11-1.29)	3,763(0.91)	938(1.71)	2.03(1.88-2.18)	221(1.11)	345(1.65)	1.58(1.31-1.91)
**Other substance use disorders**	15,825(1.51)	2,698(2.57)	1.76(1.69-1.84)	964(1.92)	944(2.39)	1.32(1.20-1.46)	1,598(0.39)	438(0.8)	2.29(2.05-2.56)	74(0.37)	137(0.66)	1.87(1.34-2.61)
**Schizophrenia**	2,388(0.23)	223(0.21)	1.01(0.88-1.16)	142(0.28)	82(0.21)	0.81(0.61-1.08)	143(0.03)	16(0.03)	1.00(0.59-1.72)	6(0.03)	<5(0.02)	0.27(0.05-1.40)
**Other psychotic disorder**	7,826(0.75)	1,004(0.96)	1.37(1.28-1.47)	436(0.87)	359(0.91)	1.10(0.95-1.27)	991(0.24)	157(0.29)	1.47(1.24-1.75)	44(0.22)	49(0.23)	1.09(0.69-1.73)
**Bipolar disorder**	12,291(1.17)	2,512(2.39)	2.01(1.93-2.10)	835(1.67)	929(2.35)	1.47(1.33-1.62)	1,762(0.42)	707(1.29)	3.36(3.07-3.67)	106(0.53)	252(1.21)	2.43(1.89-3.12)
**Stress-related disorder**	131,639(12.54)	22,041(21.00)	1.93(1.90-1.97)	7,245(14.45)	7,944(20.12)	1.63(1.57-1.70)	46,185(11.13)	10,773(19.66)	1.94(1.90-1.98)	2,392(11.98)	3,999(19.15)	1.66(1.56-1.76)

© 2026 Zhou J et al. *JAMA Network Open*.

**Eating disorder**	20,619(1.96)	3,747(3.57)	1.73(1.67-1.79)	1,509(3.01)	1,496(3.79)	1.29(1.19-1.39)	1,330(0.32)	442(0.81)	2.54(2.28-2.83)	74(0.37)	174(0.83)	2.38(1.74-3.24)
**Tobacco use disorder**	9,807(0.93)	1,264(1.20)	1.32(1.24-1.40)	546(1.09)	453(1.15)	1.10(0.96-1.25)	5,076(1.22)	686(1.25)	1.17(1.08-1.27)	218(1.09)	264(1.26)	1.29(1.05-1.59)
**ADHD**	20,385(1.94)	3,979(3.79)	2.01(1.94-2.09)	1,334(2.66)	1,507(3.82)	1.60(1.47-1.74)	2,941(0.71)	1,330(2.43)	3.55(3.32-3.80)	191(0.96)	511(2.45)	2.41(1.99-2.90)
**Autism**	7,637(0.73)	1,150(1.10)	1.60(1.50-1.70)	489(0.98)	413(1.05)	1.14(1.00-1.32)	737(0.18)	222(0.41)	2.51(2.14-2.94)	49(0.25)	88(0.42)	2.00(1.30-3.08)
**Behavioral disorder**	8,159(0.78)	1,250(1.19)	1.56(1.46-1.65)	499(1.00)	442(1.12)	1.22(1.07-1.40)	537(0.13)	183(0.33)	2.73(2.30-3.24)	36(0.18)	53(0.25)	1.48(0.88-2.50)
**Personality disorder**	16,810(1.60)	3,312(3.16)	2.01(1.94-2.09)	1,111(2.22)	1224(3.10)	1.51(1.39-1.65)	1,277(0.31)	492(0.90)	3.34(3.00-3.72)	84(0.42)	188(0.90)	2.32(1.74-3.10)

PMD = Premenstrual disorders, PSY = psychiatric disorder, OR = Odds ratio, HR = Hazard ratio.
Model: The estimates were adjusted for matching variables, country of birth, civil status, income, and educational level.

© 2026 Zhou J et al. *JAMA Network Open*.

eTable 5. Associations between (a) psychiatric disorders and risk of subsequent PMD and (b) PMD and subsequent risk of psychiatric disorders, by time of psychiatric disorders to/from PMD

**(a)Nested-case control/From psychiatric disorder to PMD**			
	No PMD	PMD	Model(OR,95%CI)[a]
**Population**			
Time window	Psychiatric disorder, n (%)	Psychiatric disorder, n (%)	
Psychiatric disorder diagnosed ≤5 years before matching	122,493(11.67)	21,012(20.02)	2.54 (2.49-2.58)
Psychiatric disorder diagnosed >5 years before matching	187,309(17.84)	29,164(27.78)	2.32 (2.28-2.36)
**Sibling**			
Time window	Psychiatric disorder, n (%)	Psychiatric disorder, n (%)	
Psychiatric disorder diagnosed ≤5 years before matching	4,310(13.10)	5,701(19.34)	2.03(1.93-2.13)
Psychiatric disorder diagnosed >5 years before matching	7,324(22.26)	8,233(27.93)	1.84(1.75-1.92)
**(b) Matched-cohort/From PMD to psychiatric disorder**			
	No PMD	PMD	Model(HR,95%CI)[a]
**Population**			
Time window	Psychiatric disorder, n (%)	Psychiatric disorder, n (%)	
≤5 years from matching to psychiatric disorder	46,684(11.3)	13,254(24.2)	2.38 (2.33-2.42)
>5 years from matching to psychiatric disorder	41,075(15.78)	6,934(24.88)	1.99 (1.93-2.04)
**Sibling**			
Time window	Psychiatric disorder, n (%)	Psychiatric disorder, n (%)	
≤5 years from matching to psychiatric disorder	1,658(12.70)	2,640(22.20)	1.96(1.83-2.09)
>5 years from matching to psychiatric disorder	231(2.53)	222(2.61)	1.13(0.91-1.40)

PMD = Premenstrual disorders, OR = Odds ratio, HR = Hazard ratio, CI = confidence interval.

Model: The estimates were adjusted for matching variables, country of birth, civil status, income, and educational level.

© 2026 Zhou J et al. *JAMA Network Open*.

eTable 6. Bidirectional associations between premenstrual disorders and psychiatric disorders, stratified by country of birth and age at matching

**(a)Nested-case control/From psychiatric disorder to PMD**					
		n	PSY, n(%)	Model (OR,95%CI)	P for interaction
**Population**					
By country of birth					<0.001
Scandinavian	No PMD	816,873	254,929(31.2)	Ref	
	PMD	90,573	43,384(47.9)	2.33(2.29-2.36)	
Others	No PMD	232,847	54,873(23.6)	Ref	
	PMD	14,399	6,792(47.2)	2.93(2.83-3.04)	
By age group					<0.001
<35 years	No PMD	486,921	147,201(30.2)	Ref	
	PMD	48,694	25,133(51.6)	2.66(2.61-2.72)	
≥ 35 years	No PMD	562,799	162,601(28.9)	Ref	
	PMD	56,278	25,043(44.5)	2.20(2.16-2.24)	
**Sibling**					
By country of birth					0.448
Scandinavian	No PMD	47,312	16,357(34.6)	Ref	
	PMD	37,603	17,556(46.7)	1.94(1.88-2.01)	
Others	No PMD	2,818	1,192(42.3)	Ref	
	PMD	1,871	1,039(55.5)	2.06(1.78-2.37)	
By age group					<0.001
<35 years	No PMD	25,603	9,500(37.1)	Ref	
	PMD	19,474	10,040(51.6)	2.13(2.04-2.22)	
≥ 35 years	No PMD	24,527	8,049(32.8)	Ref	
	PMD	20,000	8,555(42.8)	1.77(1.7-1.86)	
**(b) Matched-cohort/From PMD to psychiatric disorder**					
		N	PSY, n(%)	Model (HR,95%CI) [a]	P for interaction
**Population**					
By country of birth					<0.001
Scandinavian	No PMD	32,7642	71,193(21.7)	Ref	
	PMD	47,189	17,204(36.5)	2.20(2.16-2.24)	

© 2026 Zhou J et al. *JAMA Network Open*.

Others	No PMD	87,308	16,216(18.6)	Ref	
	PMD	7,607	2,861(37.6)	2.43(2.32-2.55)	
By age group					<0.001
<35 years	No PMD	175,603	37,208(21.2)	Ref	
	PMD	23,561	9,225(39.2)	2.43(2.37-2.49)	
≥ 35 years	No PMD	239,347	50,201(21)	Ref	
	PMD	31,235	10,840(34.7)	2.09(2.04-2.13)	
**Sibling**					
By country of birth					0.878
Scandinavian	No PMD	19,033	4,410(23.2)	Ref	
	PMD	20,047	7,186(35.8)	1.82(1.73-1.9)	
Others	No PMD	935	240(25.7)	Ref	
	PMD	832	313(37.6)	1.79(1.43-2.23)	
By age group					0.491
<35 years	No PMD	9,311	2,314(24.9)	Ref	
	PMD	9,434	3,601(38.2)	1.86(1.73-1.99)	
≥ 35 years	No PMD	10,657	2,336(21.9)	Ref	
	PMD	11,445	3,898(34.1)	1.79(1.68-1.92)	

PSY = psychiatric disorder, PMD = premenstrual disorders, OR = odds ratio, HR = hazard ratio, CI = confidence interval, Ref = reference.
Model: The estimates were adjusted for matching variables, country of birth, civil status, income, and educational level.

© 2026 Zhou J et al. *JAMA Network Open*.

eTable 7. Associations between (a) psychiatric disorders and risk of subsequent PMD, and (b) PMD and subsequent risk of psychiatric disorders, restricting to counties with primary care data

**(a)Nested-case control/From psychiatric disorder to PMD**			
	n	PSY, n(%)	Model[a],OR (95%CI)
**Population**			
No PMD	694,330	245,917(35.42)	Ref
PMD	69,433	39,551(56.96)	2.61(2.57-2.66)
**Sibling**			
No PMD	20,298	8,790(43.3)	Ref
PMD	18,916	10,838(57.3)	2.06(1.96-2.17)
**(b) Matched-cohort/From PMD to psychiatric disorder**			
	n	PSY, n(%)	Model1[a],HR (95%CI)
**Population**			
No PMD	208,348	67,038(32.18)	Ref
PMD	29,882	15,133(50.64)	2.21 (2.17-2.25)
**Sibling**			
No PMD	6,370	2,263(35.5)	Ref
PMD	5,877	3,068(52.2)	1.84(1.71-1.98)

PSY = psychiatric disorder, PMD = premenstrual disorders, OR = odds ratio, HR = hazard ratio, CI = confidence interval, Ref = reference.
[a]The estimates were adjusted for matching variables, country of birth, civil status, income, and educational level.

© 2026 Zhou J et al. *JAMA Network Open*.

eTable 8. Associations between (a) psychiatric disorders and risk of subsequent PMD, and (b) PMD and subsequent risk of psychiatric disorders, restricting to women who receive consecutive PMD diagnoses which are at least 28 days apart

**(a)Nested-case control/From psychiatric disorder to PMD**			
	n	PSY, n(%)	Model[a], OR (95%CI)
**Population**			
No PMD	487,610	142,449(29.21)	Ref
PMD	48,761	23,783(48.77)	2.33(2.28-2.38)
**Sibling**			
No PMD	15,666	5,536(35.34)	Ref
PMD	14,052	6714(47.78)	1.96(1.86-2.08)
**(b) Matched-cohort/From PMD to psychiatric disorder**			
	n	PSY, n(%)	Model 1[a], HR (95%CI)
**Population**			
No PMD	189,657	41,187(21.7)	Ref
PMD	24,978	10,065(40.3)	2.44 (2.38-2.50)
**Sibling**			
No PMD	6,102	1,440(23.6)	Ref
PMD	5,590	2,178(39)	2.08(1.92-2.26)

PSY = psychiatric disorder, PMD = premenstrual disorders, OR = odds ratio, HR = hazard ratio, CI = confidence interval, Ref = reference.
[a]The estimates were adjusted for matching variables, country of birth, civil status, income, and educational level.

© 2026 Zhou J et al. *JAMA Network Open*.

eTable 9. Bidirectional association between premenstrual disorders and any psychiatric disorder, with additional adjustment for number of outpatient visits within 6 months before the matching date

**(a)Nested-case control/From psychiatric disorder to PMD**			
	n	PSY, n(%)	Model[a], OR (95%CI)
**Population**			
No PMD	1,049,720	309,802(29.50)	Ref
PMD	104,972	50,176(47.80)	2.44(2.41-2.48)
**Sibling**			
No PMD	50,130	17,549(35.01)	Ref.
PMD	39,474	18,595(47.11)	2.00(1.94-2.07)
**(b) Matched-cohort/From PMD to psychiatric disorder**			
	n	PSY, n(%)	Model 1[a], HR (95%CI)
**Population**			
No PMD	414,950	87,409(21.06)	Ref.
PMD	54,796	20,065(36.62)	2.25(2.21-2.29)
**Sibling**			
No PMD	19,968	4,650(23.29)	Ref.
PMD	20,879	7,499(35.92)	1.84(1.76-1.92)

PSY = psychiatric disorder, PMD = premenstrual disorders, OR = odds ratio, HR = hazard ratio, CI = confidence interval, Ref = reference.
[a]The estimates were adjusted for matching variables, country of birth, civil status, income, educational level and the outpatient visit within half year before matching date.

© 2026 Zhou J et al. *JAMA Network Open*.
