# Supplementary material for: Bidirectional Association Between Premenstrual Disorders and Psychiatric Disorders
Source: JAMA Netw Open. 2026 May 8;9(5):e2611765. doi: 10.1001/jamanetworkopen.2026.11765 (PMC13156785; doi:10.1001/jamanetworkopen.2026.11765)
Supplement: Supplement 2. — Data Sharing Statement [file jamanetwopen-e2611765-s002.pdf]

Data Sharing Statement

Zhou. Bidirectional Association Between Premenstrual Disorders and Psychiatric Disorders. *JAMA Netw Open*. Published May 08, 2026. doi:10.1001/jamanetworkopen.2026.11765

Data
Data available: No

Additional Information
Explanation for why data not available: Information on accessing Swedish register data is available from the Swedish National Board of Health and Welfare (https://bestalladata.socialstyrelsen.se/, email: registerservice@socialstyrelsen.se) and Statistics Sweden (https://www.scb.se/vara-tjanster/bestall-data-och-statistik/, email: scb@scb.se). For primary care data, each county needs to be contacted individually; contact information for all counties is available at https://kliniskastudier.se/english. Data analysis codes can be shared upon request to the corresponding author.
